# Small Special Hospitals

**Published:** 1890-03-08

**Authors:** 


### Passing Topics.

### SMALL SPECIAL HOSPITALS.

We are assured by some that the current year is to be one of disaster for small special hospitals. It does not appear clearly whence the Nemesis which is to overtake them will spring, but nevertheless the notion is prevalent that these minor luminaries which so thickly stud the charitable firmament are to be tested, found wanting, and extinguished. After waiting so long, we may be excused for being sceptical of the retribution said to be at hand, but if the horoscope of the small special hospitals be indeed drawn and fate impends, few of us will fail to contemplate their disappearance with equanimity, seeing that the very first step towards rendering our hospital system sound and vigorous is to free it from those parasitic growths with which it has been long encumbered.

Whenever the time comes for taking an enlightened and comprehensive view of our hospital work, we shall be struck less by its magnitude, less even by its deficiencies, than by the unrestrained licence which permits any adventurer, who is so minded to start a hospital for his own whim or profit, and having started it, to appeal to the public to support it. In this connection the law exercises no sort of control, and it imposes no penalties. Nor does it afford the useful and regularly established hospitals, or the benevolent, any sort of protection. In vain those who act from a motive higher than mere impulse and desire that their gifts should be judiciously bestowed, look for guidance; there is none to guide.

The so-called hospital which appeals so loudly for aid, may be nothing more than an insanitary house, destitute of needful appliances, and unfit for its ostensible work. It may be officered by men who have no claim to distinction of any sort in the profession, or to possess special acquaintance with the particular maladies their hospital affects to treat. The "physician" of a special hospital has been known to fail before the examiners because of ignorance in respect of the very maladies he had appropriated as his speciality. To justify a special hospital in its existence, it is only common sense to demand special knowledge and general knowledge too. Without the former the pretensions would amount to an impertinence; if unequipped with the latter, the knowledge would be of that limited character labelled as dangerous. It is a grievous mistake to suppose that anyone can develop a speciality usefully unless the general knowledge is profound. The speciality must be something superadded. Errors in diagnosis, serious enough to the patient, cannot fail to be made by a man who sets to work to account for every condition by one set of circumstances. A specialist who is nothing more than a specialist is *ipso facto* a charlatan, and no less so because he may be a successful one. To be a meritorious specialist, a man must be alive to all the possibilities of disease, for he it is who is most often called to decide upon an obscure case. As a matter of fact, the self-appointed "physician" of a petty hospital is usually a man below the standard all round, and no better fitted to discover and deal with a deep seated malady than a village blacksmith is to rebuild a shattered steam engine. At a hospital many doubtful and difficult cases may present themselves in the course of a single day, and must be dealt with rapidly, and if we reflect upon this fact in connection with the ill-founded pretensions of some of the small special hospitals, it will be easy to see that the danger incurred by the patient calls for at least as much attention as the imposition suffered by the donor. There is too much tendency to consider the financial side only. That is important and without doubt it calls for grave protest that alms for lack of which better institutions languish, are intercepted by places of predatory instincts whose existence is not justified by the performance of any good work, but if it were possible to learn something of the secrets of the consulting-room, we should find, we fear, but too much additional reason for desiring the advent of that long-deferred time when medical charity will be purged of its excesses and its impostures.

Agricola.

